# Explaining the Diffusion of Project ECHO

**DOI:** 10.1186/s43058-025-00778-x

**Published:** 2025-08-19

**Authors:** James W. Dearing, R. Sam Larson

**Affiliations:** 1 Department of Communication, Michigan State University, 473 CAS Building, 410 Wilson Road, East Lansing, MI 48824-1212 USA; 2 Diffusion Associates, 606 Marshall Street, East Lansing, MI 48823 USA

**Keywords:** Diffusion of innovations, Project ECHO, Telementoring

## Abstract

Project Extension for Community Healthcare Outcomes (Project ECHO), a telementoring intervention in which medical specialists share knowledge with medical generalists, has spread to many sites during its 22 years, reaching providers in nearly 200 countries. Based on our familiarity with the ECHO Institute, its practices, and our work with ECHO implementers at many sites, we explain the diffusion of this telehealth intervention in which medical specialists and generalist providers mentor each other in delivering specialty care to patients. We find the diffusion of Project ECHO to be well-accounted for by traditional factors including the perceived attributes of the ECHO model, the status of prominent ECHO adopters, and the inter-organizational environment within which the model arose. We also identify aspects of the ECHO model that have not always figured prominently in studies of diffusion but likely stimulated diffusion in this case. These include charismatic leadership, model elasticity, optional evaluation, and bounded elasticity. The Project ECHO experience can inform the decisions by proponents of other health care innovations to accelerate and broaden diffusion.

**Supplementary Information:**

The online version contains supplementary material available at 10.1186/s43058-025-00778-x.

Contributions to the literature

- A contrast is drawn between an intervention whose advocates prize adoption versus interventions in which implementation and sustainment are more highly valued.
- We identify factors that explain the diffusion of a widely adopted intervention while introducing the concepts of principle-based innovations, expectant fidelity, and bounded elasticity.
- A successfully disruptive innovation was far from being a radical innovation in medical telementoring.

## Background

The diffusion of innovations research and practice paradigm has been applied many times in post-hoc analyses to understand why some innovations spread while others do not [1]. Diffusion involves a new practice, program, policy, or technology that is communicated over time among the members of a social system.

**Fig. 1:**
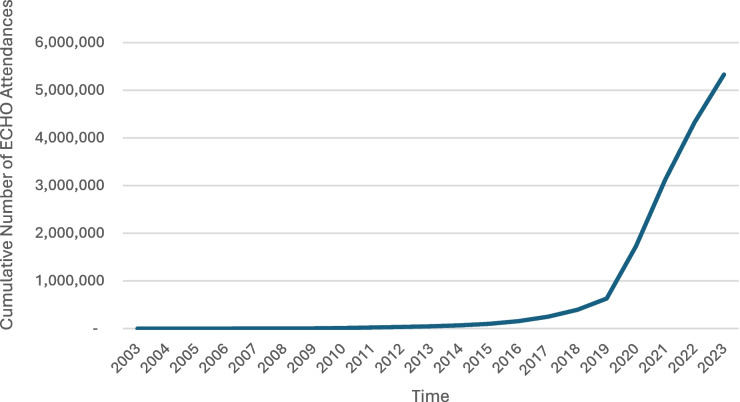
Cumulative number of attendances in Project ECHO sessions

Since a considerable majority of innovations do not achieve the rapidity nor the reach desired by their proponents, the experiences of innovations that do so can be instructive for health care innovation teams, funders, and policy makers. The telementoring intervention Project Extension for Community Healthcare Outcomes (Project ECHO) [2] is one such case, begun in 2003 in New Mexico to teach and share hepatitis C care with rural providers in that state. ECHO has had more than 1 million attendances annually since 2020, involving providers from more than 200 countries in 2023 when 1,275 new multi-session online programs were launched from 220 new hubs [3] at academic medical centers, hospitals, health systems, and universities (Fig. 1). Government agencies including the U.S. Department of Defense and the U.S. National Cancer Institute host ECHO programs as do medical societies and nonprofits.

What explains the diffusion of Project ECHO? In 2018, we were asked to study the ECHO model by the Robert Wood Johnson Foundation which had supported ECHO for some years. A succession of grants from the foundation enabled us to meet, observe, and collaborate with ECHO leaders across North America (the Appendix describes our methodology). Ostensibly studying the model’s implementation, as students of diffusion we additionally attended to how the model was taught, how enthusiasm built, how new hubs and programs arose, and relations between the ECHO Institute in Albuquerque and what many implementers in the field referred to as “the movement” of far-flung autonomous ECHO hubs across the globe.

### Traditional explanatory variables and ECHO diffusion

Researchers have identified three general sets of explanatory variables that often account for adoption decisions and, hence, innovation diffusion: Perceived attributes of an innovation; the effect that prior adopters can have on later adopters; and the context into which an innovation is launched such as when it is introduced and for which problem the innovation is framed as a solution. When positive, the convergence of these factors can propel an innovation up the cumulative S-shaped diffusion curve [4].

The ECHO model benefitted from all three of these sets of variables. In terms of how it was perceived by adopters, ECHO was easily made compatible with current practices, it was simple to understand and implement, cost was minimal since there was no licensing fee and adopting organizations already had the necessary technology and substantive expertise, there was evidence that the model had, at a minimum, positive effects on rural care providers, a small team could try hosting an ECHO program without loss of resources if they changed their minds, and how others implemented the sessions was readily observable. When visitors from academic medical centers and other types of health care organizations traveled to the ECHO Institute in the University of New Mexico School of Medicine for training, they saw that what they needed back home to begin an ECHO they already had: An internet connection, a small videoconference room, medical expertise, and email addresses of rural or urban providers of care for underserved communities. ECHO had no licensing fee, no required protocols, no minimum data set mandated for evaluative purposes. A staff member could be the organizational point person for multiple ECHO programs — one of which might address behavioral health, another cancer survivorship, a third chronic pain management — and still be responsible for other duties. Administrative staff and specialists’ time might be funded for each program by addressing an acknowledged rural or urban need.

Partly because of the in-person training, how the model could be enacted was easily observable. Indeed, the model was inherently visible with everyone in each ECHO program on screen together (the Institute’s license with Zoom allows free use by implementing sites). Every component of ECHO — the use of communication technology, the sharing of best practices, case-based learning, and especially the modeling of ECHO-style group facilitation so that “all teach, all learn” — was familiar, an easy lift for someone wanting to start an ECHO program. Trainees watched how ECHO program sessions ran and came away convinced that if they recruited the facilitator-specialists whose demeanor created a sense of psychological safety in the group [5], they could replicate ECHO’s secret sauce of specialists listening carefully and asking questions of general practitioners, a modeling of professional humility [6]. Trainees’ self-efficacy was reinforced by the community of practitioners[7] they were now a part of, in relationships forged at trainings and online together in ECHO program sessions.

Secondly, in terms of the importance of early adopters on the decisions of later adopters, the early adopters of Project ECHO, impressed by prominent publications of effects in 2007 [8] and 2011 [9, 10] included high-status institutions: MD Anderson Cancer Center, University of Chicago, American Academy of Pediatrics, U.S. Department of Veterans Affairs, and the U.S. Centers for Disease Control and Prevention. Thereafter, demand for ECHO training grew. The signaling by influential early adopting organizations can be a make-or-break moment in a diffusion process. Because their actions are noted by others, the decisions by early adopting opinion leading organizations are of critical importance for an innovation such as Project ECHO. Swelling interest led the ECHO Institute to designate large ECHO hubs as “superhubs” so that a training bottleneck could be avoided by sharing the burden of training with large ECHO operations such as those at the University of Missouri and thus not impede growth.

The third traditional explanation for innovation diffusion (and more commonly, lack thereof) is the inter-organizational environment of external conditions. When and how an innovation is launched can dramatically affect diffusion. Innovations with a positive set of attributes and that benefit from the credibility of early-in-time investors, proponents, and adopters can still fail to diffuse without an accommodating context [11]. Whereas Zoom was novel in ECHO’s early years, COVID-19 made all of us fluent Zoom users. And it wasn’t just that the pandemic normalized online meeting and learning; it was that Project ECHO leadership and the model’s thousands of implementing sites were prepared to respond to a crisis. The infrastructure was in place and operational, the staffing and training done. The ECHO model was selected by the U.S. Agency for Healthcare Research and Quality for its $237 million ECHO National Nursing Home COVID-19 Action Network for training tens of thousands of nursing home staff as soon as possible. Use of ECHO increased more than fourfold from 2019 to 2020, exceeding its annual growth rates prior to COVID-19.

### Other reasons accounting for the diffusion of Project ECHO

#### Charismatic leadership

Identification with a passionate and credible social entrepreneur can be a boon to an innovation, bringing a degree of star quality to an innovation. Sanjeev Arora, staff gastroenterologist at University of New Mexico Hospital and founder and executive director of Project ECHO, has been indefatigable in demonstrating session facilitation for trainees, in presenting the model to funders, in promoting the model to potential partners, being spokesperson for the model in media coverage, and in encouraging Federal support. While strong identification of a leader with an innovation can eventually limit diffusion [12], a dedicated and energetic social entrepreneur can tell the story of an innovation in a memorable humanistic way.

#### Model elasticity

Innovations that are amenable to adaptation more readily diffuse [13] than those innovations that are not. ECHO is a highly elastic model. Topically, the ECHO model can be considered an agnostic platform. ECHO programs have been launched to address hundreds of health conditions and procedures. Various types of organizations host ECHO hubs. Operations can thrive in an organization’s continuing education division, in an academic department, in a hospital clinic. Staff can host one program or many. An ECHO program can be led by one specialist or a panel of interprofessional specialists. A program can have a set number of sessions or go on indefinitely. Session length can vary as can frequency. The science behind an ECHO topic can be well-established as in ECHO programs focused on endocrinology, enabling concrete suggestions, or multi-faceted and highly contextualized as in behavioral health. What is less elastic is the number of participating general practitioners. ECHO sessions are not webinars; ideally, they are small group discussions of 8–20 individuals in which”all teach, all learn”. A well-run ECHO program levels the playing field for generalists and specialists with no source of knowledge privileged over others. Learning by generalists [14] is the objective, but specialists can learn, too in this participatory adult education model [15].

#### Optional evaluation

Project ECHO eventually provided implementing sites with a data management and evaluation system, but use, let alone consistent use, while encouraged, is voluntary. The collection and monitoring of data is a part of the ECHO model, but there is no minimum data set of variables to be measured. Many staff at ECHO’s thousands of distributed sites do collect data about the number of participants, program completion, satisfaction, knowledge gain, sense of community, and intention to use what has been learned. Patient outcome data are still unusual metrics for ECHO programs. Part of the difficulty of institutionalizing the collection of a set of standard measures is the diversity of ECHO programs. Some ECHOs focus on clinical management or operational challenges, not the delivery of specialty care. Some ECHO participant health providers see patients; others, like social workers and community health workers, see clients or community members. The elasticity of the model that has facilitated its diffusion has curtailed the collection of data for comparison. Are brief time-limited programs superior to ongoing open-ended session formats? Does the model work especially well for imparting knowledge to generalist providers about certain diseases but not other diseases? Not being able to assess model performance across its many implementing sites has greatly hampered the extent to which knowledge claims can be made about ECHO. While ECHO can be said to improve access to specialty care—and maybe that’s enough for patient populations that have traditionally lacked access—other questions about patient impact have less definitive answers. Innovation diffusion is usually measured as cumulative adoptions over time. This is a measure of quantity as in the number of hubs or programs or participants or attendances, not quality as expressed in implementation fidelity, health outcomes, or population impact.

We have come to understand Project ECHO as a *principle-based innovation* in which adopters are trained through social modeling to understand how to enact one principle (“all teach, all learn”) through the use of several intervention components as they see fit to realize the principle. Adaptation is encouraged but within reason, as communicated to trainees through their training observations and as guests in actual program sessions.

#### Prioritization of adoption over implementation and sustainment

When a program has a goal of helping one billion people by the end of 2025 [16], growth is the means to that end. This goal prompted continual expansion of the model: more health conditions being addressed, more hubs being founded, more programs launched, more participating providers, all in pursuit of the goal to benefit more patients. Attention by the ECHO Institute was focused on promotion, spreading the word, training and supporting new hubs and programs, and of course funding to support more staff at the Institute to carry out growth-oriented activities. Time and attention from the ECHO Institute was not lavished on long-time sites, even those exemplars where ECHO was prominent, wide-ranging, well-funded, and where outcome data were being collected and used.

The ECHO Institute follows an approach to implementation of *expectant fidelity*. Whereas implementation fidelity is very often measured by comparing how implementers are trained with how they subsequently enact an intervention, with ECHO there is no annual or recurring assessment overseen by the Institute or a confederate such as a training superhub. Since a principle-based innovation is intended to allow for and even encourage adaptation, there is only an expectation that each of an intervention’s components will be put into practice without rigid compliance of fidelity.

Some innovations are robust in how they can be implemented and can be used to good effect in a multitude of ways. Among our ECHO collaborators—lead staff we had recruited to partner in practice-based research with us—there was a strong sense of self-policing and awareness of what was—and wasn’t—an ECHO program. We came to understand this expert awareness of theirs as a *bounded elasticity* for each of the ECHO components. Each component could be adapted to fit one’s circumstances, but only so far.

## Discussion

The diffusion of innovations paradigm as applied to the study of health innovations includes many concepts. The three factor-sets of explanatory variables that often account for diffusion—the perceived attributes of an innovation, the effect of early adopters on subsequent others, and the inter-organizational environmental context into which an innovation is launched—have been established through the inductive study of many innovations in post-hoc fashion as inquiries to explain both successful and failed diffusion [17]. These studies, in turn, led to modeling and simulation work [18] and large-scale diffusion experiments [19] that helped clarify the importance of these factors to people and organizations making adoption decisions.

As a health innovation, Project ECHO’s facilitated style of multi-directional group engagement hit on all cylinders from a diffusion perspective if one uses adoption as a main dependent variable as so many diffusion studies have done. As a telementoring model, ECHO is seen as having plentiful advantages, it is in regular use at many illustrious institutions, it offers a means to improve health equity by broadening access, and its chameleon-like adaptability to the needs and capacities of adopting health care organizations make decisions to try it easy and low-risk. ECHO was the right model at the right time for the right problem.

What took back seat in this growth-focused diffusion approach by the ECHO Institute at the University of New Mexico was attention to and support for implementation and sustainment for the thousands of ECHO hubs and programs already onboard. Despite the existence of virtual collaboratives hosted by the Institute, the leaders and staff we interviewed or worked with felt isolated. For them it was challenging to find out what other ECHO operations were doing and how they resolved routine, persistent operational challenges such as: How should we elicit patient cases from community-based providers for discussions? With which types of funders have implementers had the most success? What works best for recruiting and continuing with remote community-based providers? Which appeals have proved most effective for identifying and engaging organizational champions to advocate for hard money positions? What can medical leaders do to foster the “all teach, all learn” virtual group dynamic? How is program evaluation best performed? Answers to questions such as these—the nuts and bolts of health program implementation and sustainment—have not been the ECHO Institute emphasis even while they have been emphases of certain well-funded ECHO implementing sites. A driving focus on growth—adoptions of ECHO by more sites to benefit more disadvantaged patients in rural and inner city America and in India and African countries—left implementation and ensuing institutionalization up to the many adopting sites, a frustration reported to us by our 25 collaborating ECHO Implementation Fellows. One of our deliverables, produced with the Fellows, provides answers to these and related questions and is freely available for implementing staff [20].

Was ECHO a radical innovation? Innovations radically different from standard practice face more and higher barriers to adoption than incremental innovations [21]. ECHO was very different from how physicians engaged in continuing medical education, online learning, peer education, and communities of practice. The model struck providers as something novel due to its pragmatic combination of communication technology, best practice didactics, and case-based learning. These components themselves were not novel. They have been central to medical education for a long time. What was different about ECHO was its combination of these components in one intervention.

If Project ECHO is not best understood as a radical “really new” innovation, it does fit the conditions of being a disruptive one [22]. ECHO both broadened access to specialty care and reduced the behavioral and monetary costs of that access.

From other studies of ours [23], we have identified other health innovations that are well-suited to a growth-focused diffusion approach as enacted by Project ECHO proponents. These innovations share a presumed robustness in implementation that allows the disseminating change agencies to largely absolve themselves of attention to implementation so that they can focus limited resources on attaining greater reach. While an adoption emphasis is inappropriate for innovations that must be implemented with strict fidelity, such an emphasis may help to achieve change agency goals for principle-based innovations when fidelity is treated as expectant as with Project ECHO.

## Conclusions

We investigated a health care innovation for which its change agency enacted a no-holds barred diffusion strategy focused on adoption rather than implementation and sustainment. The change agency objective was to grow as quickly as possible the number of locations deploying this innovation—and grow it did. We used three sets of explanatory variables from diffusion of innovation theory—the attributes of the innovation, the ways in which opinion-leading early adopters affect the decisions of subsequent others, and the timing and framing of the innovation’s introduction—to explain its many adoptions, along with the identification of several other contributing factors to the diffusion of Project ECHO.

## Supplementary Information

Supplementary Material 1.

## Data Availability

Not applicable.

## Abbreviation

ECHO: Project Extension for Community Healthcare Outcomes
