# Supplementary material for: Explaining the Diffusion of Project ECHO
Source: Implement Sci Commun. 2025 Aug 19;6:88. doi: 10.1186/s43058-025-00778-x (PMC12363020; doi:10.1186/s43058-025-00778-x)
Supplement: Supplementary file 1 — Supplementary Material 1. [file 43058_2025_778_MOESM1_ESM.docx]

**Explaining the Diffusion of Project ECHO**

**Appendix**

**Methodology**

We learned about Project ECHO through several means via three grants from the Robert Wood Johnson Foundation.

The first body of work was a scoping review and research agenda conducted in 2018-2019. Published studies and reviews about Project ECHO were identified by referring to a bibliography maintained on the University of New Mexico School of Medicine Project ECHO website and a more detailed list of publications shared with the authors by Project ECHO leadership, as well as an internet search for other reports and publications relevant to the scoping review purpose. As a result, we identified 129 peer-reviewed publications that either reported outcomes of, or reviewed, Project ECHO. Publications appeared between 2007 and 2018 and had been cited more than 1900 times.

Once the full database of articles had been established, each publication was categorized based on the processes or outcomes studied, if any. Outcomes had already been coded by Project ECHO staff in the detailed list of publications so their codes were retained for the purposes of our review. We used this categorization to identify which articles were relevant for assessing the efficacy and effectiveness of Project ECHO for patient access to care, patient outcomes, patient engagement, patient satisfaction, and provider outcomes of several types.

A second grant for 2020-2023 enabled us to recruit a large research-practice team of four behavioral researchers and 25 Project ECHO administrative staff to study ECHO hub and program implementation at 34 ECHO hubs and across 62 ECHO programs in North America. An ECHO hub is an organizational unit that coordinates and offers multi-session ECHO programs. We studied hubs perceived as influential within the Project ECHO community, defined as hubs that leaders of other hubs might look to for advice or ideas to improve their own operations. To determine which hubs were influential, we met with Sanjeev Arora, MD, founder of Project ECHO at the ECHO Institute in New Mexico, and asked him to generate an initial list of influential hubs. To this list, we added hubs whose leaders had made presentations at international MetaECHO conferences in 2014, 2016, 2017 and 2019, and published peer-reviewed journal articles about ECHO through 2018. We limited inclusion to hubs that had been in operation for at least one year. This process yielded 57 influential hubs.

With a letter of introduction from Dr. Arora, we contacted leaders of each influential hub, inviting them to be a study site. After repeated outreach efforts, 34 hubs agreed to participate in this study. Of the 34 hubs, 17 were affiliated with universities, 11 were in clinical or medical centers, and six were nonprofit or government affiliated organizations. So that we could learn about ECHO program implementation as well as hub implementation, we then selected programs within each hub to participate in the study. We wanted programs with a focus on health care that had completed at least one multi-session cycle within the prior year. In six of the hubs, only one ECHO program met these inclusion criteria; from each of the remaining 28 hubs we selected two ECHO programs that differed from each other in terms of health topic or other program parameters including if the program was facilitated by one specialist or a panel of specialists, program maturity, and the number of sessions per program. By selecting “most different” ECHO programs within hubs we maximized differences and then in analysis attended to similarities in outcomes. We framed our work as participatory research. That is, some of the people whose work was the subject of the research actively took part in the research. Participatory research engages community stakeholders to work alongside researchers in all stages of the research process, from problem identification and developing research questions to participating in interviews, analysis, and writing. We recognized that those working in ECHO hubs and with ECHO programs would have lived experience in ECHO implementation that would deepen our knowledge and improve the study. At 25 of the 34 ECHO hub sites, we recruited individuals to participate in this study as Implementation Fellows. Fellows were recruited by asking hub leaders to identify the day-to-day administrative leaders of ECHO work at their site. We reached out to these administrative leaders and told them about the study, clarified the role of an Implementation Fellow, and discussed time commitment as well as the potential benefits of participating as an Implementation Fellow. To offset the costs of participation, we provided a stipend to each of the 25 ECHO hubs. Fellows attended six workshops led by the authors which focused on implementation science, qualitative interviewing, case study analysis, and cultural competence. In between sessions we worked with Fellows to design an online survey and an interview protocol. For each hub and program, we invited two to four people who were instrumental in leading, organizing, and implementing Project ECHO work to participate in a group interview. With the Fellows, we conducted 96 group interviews with 160 unique respondents via Zoom from September 2020 through October 2021. Interviews lasted from 60-90 minutes and were recorded and transcribed. Group interviews followed a structured protocol approved by the Western Institutional Review Board. Consent statements were shared with all respondents in advance of the interview and read prior to commencing with the interview. Respondents gave verbal consent which was recorded. Some respondents engaged in both a hub and program interview. We did not interview community-based program participants, only ECHO staff and medical specialists. Most of the respondents held administrative positions related to ECHO with titles such as center director, program manager, curriculum developer, and coordinator. Sixty respondents were specialists with medical degrees (MD, DO). We asked all respondents the same open-ended questions, designed to learn how they defined and operationalized the principle of “all teach, all learn”, origin stories about adoption, as well as their perspectives on factors related to implementation and sustainment. A series of close-ended items were asked about operational, staffing, and funding bases of hubs and programs. Four coders who had taken part in the interviews independently reviewed each coded transcript. Two coders then met and reviewed coded text to discuss and resolve differences. Our next step was to conduct an inductive multi-phase thematic analysis. We began this process by having two coders individually review each identified text section and inductively assign it an initial code so that text sections could be categorized. Coders then met to review codes, looking for similarities among codes, combining codes, and developing and defining new codes. This process was repeated one additional time when the coders reached an intercoder reliability of .80. All differences were discussed and resolved during the final coding.

A third grant was used by the project team to create and offer a multi-session ECHO program designed to accelerate organizational learning about program implementation and sustainment by ECHO staff dispersed at various locations. Sessions focused on best practices in project management, staffing, training of new staff, champions, internal funding, external funding, quality improvement, program fidelity, and program evaluation. The program was fully subscribed and reoffered.
